# Diagnosis of Acute Dengue Virus Infection Using Enzyme-Linked Immunosorbent Assay and Real-Time PCR

**DOI:** 10.1155/2023/3995366

**Published:** 2023-05-22

**Authors:** Gohar Iqbal, Hasnain Javed, Faiz Ahmed Raza, Umar Farooq Gohar, Warda Fatima, Mohsin Khurshid

**Affiliations:** ^1^Provincial Public Health Reference Laboratory, Punjab AIDS Control Program, Primary and Secondary Healthcare Department, Government of the Punjab, Lahore, Pakistan; ^2^Health Research Institute, National Institutes of Health Research Centre, King Edward Medical University, Lahore, Pakistan; ^3^Institute of Industrial Biotechnology, Government College University Lahore, Lahore, Pakistan; ^4^Institute of Microbiology and Molecular Genetics, University of the Punjab, Lahore, Pakistan; ^5^Institute of Microbiology, Government College University Faisalabad, Faisalabad, Pakistan

## Abstract

Dengue fever is a viral infection caused by the dengue virus and is a growing concern for public health worldwide, particularly in tropical and subtropical regions. This study aimed to assess the diagnostic accuracy of a commercially available NS1 ELISA kit for dengue fever in Pakistan using multiplex qRT-PCR as the gold standard. The study recruited 1236 suspected cases of dengue fever admitted to public sector hospitals in Lahore, Pakistan. Of the suspected cases, 610 (49.3%) were confirmed positive for DENV infection through qRT-PCR, with all four serotypes detected. DENV-2 was the most prevalent serotype, detected in 95.7% of cases. The NS1 ELISA kit detected 71.1% of the positive cases. However, the diagnostic accuracy of the NS1 ELISA kit was found to be only 64.89%. Of the 610 confirmed cases, 68% were male and 32% were female, with a median age of 30 years. Dengue fever was diagnosed in 91.8% of cases, while 8.2% were diagnosed with dengue hemorrhagic fever (DHF). DHF patients had a higher prevalence of abdominal pain, hemorrhagic manifestations, and thrombocytopenia. The cocirculation of all four DENV serotypes in Lahore is concerning and could lead to more severe forms of the disease, such as DHF or dengue shock syndrome, in the future. The study highlights the low diagnostic accuracy of commercially available NS1 ELISA kits and emphasizes the importance of using molecular methods to confirm acute dengue infections. Given the increasing prevalence of dengue fever in developing countries like Pakistan, more accurate and reliable diagnostic tools are needed for effective disease management and control.

## 1. Introduction

Globally, the incidence of dengue fever has increased dramatically in recent decades and has become a public health threat of grave concern. About 390 million people contract dengue virus (DENV) infection yearly in more than 128 countries, resulting in 40,000 deaths annually [1, 2]. Among them, 92 million develop symptoms of variable intensity, ranging from self-limiting dengue fever (DF) to life-threatening dengue hemorrhagic fever (DHF) and dengue shock syndrome (DSS). DENV is a positively sensed nonsegmented RNA *Flavivirus* belonging to the family *Flaviviridae*. The virus is transmitted chiefly by *Aedes aegypti* (*Ae. aegypti*) and *Ae. albopictus* mosquitoes. The virus has four antigenically distinct serotypes, including DENV-1, DENV-2, DENV-3, and DENV-4, with variable geographical distribution around the globe [3, 4]. Primary infection with one of the DENV serotypes confers long-lasting immunity against the respective serotype. In contrast, the secondary infection with the heterologous DENV serotype might result in a more severe form of the disease (DHF/DSS) through a less understood pathway of antibody-dependent enhancement (ADE) [5]. Currently, no truly safe and effective antiviral agent or vaccine is available against the DENV infection. In the absence of approved therapy, fluid replacement therapy, the use of the antipyretic drug acetaminophen, and other symptomatic treatments remained the mainstay of therapy for DENV infection [2].

Although no part of the world is free from dengue, it is more common in the tropical and subtropical regions where Pakistan is also located. In these regions, cases of dengue fever start to emerge rapidly after monsoon rainfall each year [6, 7]. Cases of DF were reported for the first time in Lahore, Pakistan, in 1968 [8], and several outbreaks have been reported afterward [9]. The first significant outbreak of DHF occurred in 1994 in Karachi, followed by another in 2005. In 2011, dengue spread to various regions of Punjab and caused morbidity and mortality on a larger scale. Now DF has become endemic, with an annual recurrence in different cities in Pakistan [10].

Early and accurate diagnosis of dengue is the primary clinical goal for evidence-based management, vector surveillance, and control measures. Currently, commercially available diagnostic techniques mainly include the detection of DENV antigens or antibodies through rapid immunochromatography (ICT) devices, enzyme-linked immunosorbent assay (ELISA), and detection of viral nucleic acid through reverse transcriptase polymerase chain reaction (RT-PCR) [11]. During the acute phase of dengue fever (up to 6 days after the onset of illness), isolation of the DENV and nucleic acid detection are the ideal methods for diagnosing infection [12]. Viral nucleic acid detection is much easier and requires less expertise, competence, and infrastructure than viral isolation. Viral nucleic acid detection through RT-PCR is more sensitive and specific, with a faster turnaround than viral isolation methods. The development of multiplex real-time RT-PCR further enabled the detection of all four DENV serotypes in a single reaction tube, thus making it a highly suitable technique for rapid diagnosis of DENV infection and large-scale surveillance studies [13].

On the other hand, viral antigen detection methods rely on the detection of nonstructural protein-1 (NS1) antigens in the patient's sera. The NS1 antigen is a glycoprotein required for the replication and viability of DENV. The NS1 antigen is secreted by mammalian cells in high concentrations, becomes detectable as early as day 1 of the onset of illness, and remains detectable for up to 5-6 days [13]. Therefore, it is valid for the early detection of DENV infection in both primary and secondary infections. Many commercial ELISA kits are available to detect DENV NS-1 antigen with high sensitivity and specificity, but these kits do not differentiate among various DENV serotypes. The current study compared the diagnostic accuracy of commercial ELISA and qRT-PCR kits to diagnose DENV infection in the patient's sera.

## 2. Materials and Methods

### 2.1. Ethical Considerations

Ethical clearance for this study was taken from Institutional Review Committee, Punjab AIDS control program (PACP-IRC). Written informed consent was taken from all the study participants or their legal guardians in the case of minors.

### 2.2. Study Site and Sampling

This prospective laboratory study was conducted during peak dengue season over a period of 6 months from August 2021 to January 2022 (*n* = 1236) in the Punjab Public Health Reference Laboratory (PPHRL) and Punjab Aids Control Program (PACP) Associated with the Primary and Secondary Health Care Government of Punjab, Lahore. The samples were collected from tertiary care hospitals, including Mayo Hospital and Sir Ganga Ram Hospital, Lahore, Pakistan.

Clinically suspected cases of dengue presenting with fever from 1 to 9 days, along with three more symptoms of dengue fever, including headache, myalgia/arthralgia, retro-orbital pain, rash, abdominal pain, and hemorrhagic manifestations, were recruited in this study. In contrast, patients who presented with a fever for >10 days and had a fever with the associated focal source of infection (e.g., otitis media, pneumonia, and meningitis), chronic illness, including anemia, and unstable vital signs were excluded from the study. Dengue cases were classified into dengue fever (DF) and dengue hemorrhagic fever (DHF) according to the World Health Organization's (WHO) classification and case definition [14].

A 3–5 mL blood sample was collected from each participant under aseptic conditions. The sample was divided into an EDTA vial for a complete blood picture and a gel tube for serum separation. The demographic and clinical characteristics of each participant were noted on the data collection tool. The samples were transferred to the laboratory for further testing. The serum was separated by centrifugation at 5000 rpm for 10 minutes and stored at −80°C for further processing.

### 2.3. Complete Blood Picture

Whole blood collected in an EDTA vial was used to determine a complete blood picture using an automated hematology analyzer (Sysmex KX-21, Japan). Hematology low (Eightcheck® 3WP-L, Sysmex) and normal (Eightcheck® 3WP-N, Sysmex) controls were used to check the performance and calibration of the hematology analyzer. Reference intervals for the interpretation of CBC results were adopted from the previously published criteria [15]. A leukocyte count of less than 4500/mm^3^ was defined as leucopenia. A platelet count of less than 150,000/mm^3^ of blood was defined as thrombocytopenia. A hematocrit (HCT) of more than 54 and 47 mL/dL in males and females was considered elevated. A hematocrit of more than 49 mL/dL was taken as elevated in children.

### 2.4. DENV NS1 Antigen Detection Using ELISA

NS1 antigen ELISA was performed on all sera samples using a commercially available Dengue Ag RecombiLISA kit (CTK Biotech USA, catalog #E0312). The kit has a sensitivity of 100% and a specificity of 97.8%. The manufacturer's instructions were followed for performing the ELISA, and each batch was evaluated using positive and negative controls in duplication and interpreting the results as reactive, nonreactive, and equivocal. Washings were performed using an ELISA microtiter plate washer (Combiwash, Human Gmbh, Ref. No. 18460). The optical density (OD) was measured at 450 nm using an ELISA reader (HumaReader HS, Human Gmbh, Ref. No. 16670). A cut-off greater than 0.3 indicated a positive sample, and a cut-off less than 0.3 indicated a negative sample.

### 2.5. RNA Extraction and Real-Time PCR (qPCR)

DENV RNA was extracted from serum samples using a commercial nucleic acid extraction kit (Zybio, China) using the manufacturer's protocol. After extraction, DENV RNA was amplified using a real-time reverse transcription PCR kit (Bosphore® Dengue virus Genotyping kit v1, Catalog # ABDEG3) to detect all four DENV serotypes in a single reaction tube. The PCR kit was used with the QuantStudio real-time PCR system (Thermo Fischer Scientific USA). The kit has a high analytical sensitivity to detect as low as 500 copies/mL of the sample. The kit was stored at −20°C before use, and components were placed on a cooling block during preparation for PCR. Internal and positive controls were used to determine the assay's performance.

### 2.6. Statistical Analysis

The data were entered and analyzed in Microsoft Excel and Statistical Package for Social Sciences (SPSS). A histogram was plotted to determine the distribution of the participants into different age groups. Sensitivity, specificity, positive predictive value (PPV), negative predictive values (NPVs), and concordance were calculated to determine the test's diagnostic accuracy.

## 3. Results

A total of 1236 suspected dengue cases reported to public sector hospitals in Lahore were recruited in this study. The study population included 820 (66.3%) males and 416 (33.6%) females. The median age of the participants was 30 years (range = 1 to 90 years). There were 106 (8.6%) children (≤16 years old) and 1130 (91.4%) adults.

**Figure 1:**
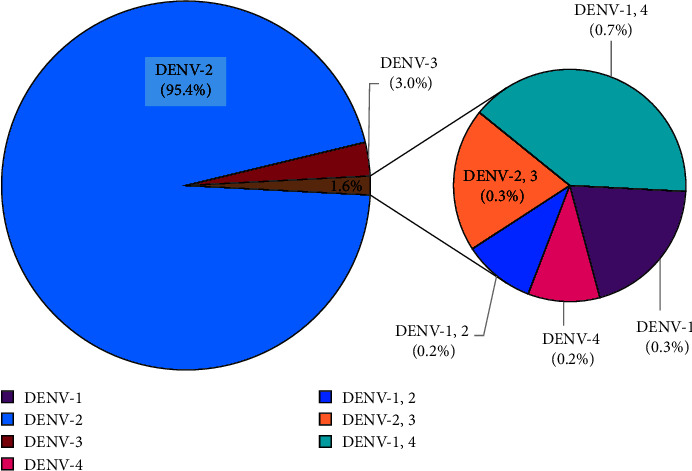
Dengue virus (DENV) serotypes circulating in Lahore in 2021.

Quantitative multiplex qRT-PCR was conducted to determine the circulating DENV serotypes in Lahore in 2021. Among 1236 cases, 610 (49.3%) were positive for DENV infection, and all four serotypes were detected in Lahore. Among them, 7 (1.2%) cases were positive for DENV-1, 584 (95.7%) for DENV-2, 20 (3.3%) for DENV-3, and 5 (0.8%) were positive for the DENV-4 serotype. In addition, concurrent infections with two different serotypes (DENV-1 and DENV-2; DENV-1 and DENV-4; or DENV-2 and DENV-3) were also detected in 7 (1.2%) patients. Notably, only 1 (0.16%) case with DENV-1 and DENV-4 concurrent infections developed DHF.  Figure 1 summarizes the frequencies of each DENV serotype circuiting in Lahore in 2021.

ELISA was conducted to detect DENV NS1 antigen in the patient's sera. Among a total of 1236 cases, 879 (71.1%) were positive for DENV NS1 antigen, including 602 (73.4%) males and 277 (66.5%) females. The diagnostic accuracy of ELISA NS1 was determined using qRT-PCR as a gold standard. The results indicated that 353 (28.5%) cases were false positive, 81 (6.6%) were false negative, 529 (42.8%) were true positive, and 104 (8.4%) were true negative. The sensitivity of the ELISA kit was 86.72% (95% CI = 83.77% to 89.31%), and the specificity was 43.61% (95% CI = 39.68% to 47.60%). The positive predictive value (PPV) was 59.98% (95% CI = 58.15% to 61.78%), and the negative predictive value was 77.12% (95% CI = 72.98% to 80.79%). The final diagnostic accuracy of ELISA to detect dengue NS1 antigen was 64.89% (95% CI = 62.15% to 67.55%). We found 529 samples positive by both tests, and 273 were negative by both tests. The concordance was 64.8%.  Table 1 shows the sensitivity, specificity, positive predictive value, negative predictive value, and accuracy of ELISA.

**Figure 2:**
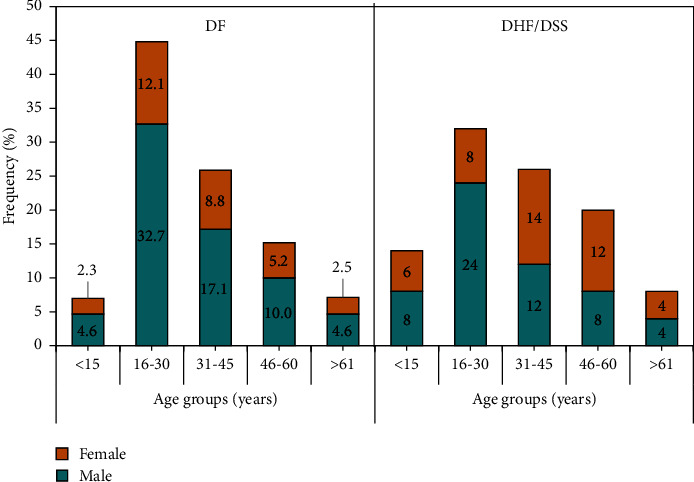
Gender-wise distribution of dengue cases among different age groups according to disease severity.

The median age of 610 PCR-confirmed dengue cases was 30 years. The highest number (*n* = 267, 43.8%) of participants belonged to the age group of 16 to 30 years, followed by 31 to 45 years (*n* = 158, 25.9%) and 46 to 60 years (*n* = 95, 15.6%) (Figure 2). The lowest number of patients belonged to the age group of >61 years (*n* = 44, 7.2%). Among clinical signs and symptoms, fever was detected in 610 (100%) cases, followed by retro-orbital pain in 436 (71.5%), abdominal pain in 435 (71.3%), and myalgia/arthralgia in 418 (68.5%) cases. Various types of hemorrhages were reported in 252 (41.3%) cases. Among these patients, thrombocytopenia was observed in 430 (70.5%), while leucopenia was observed among 290 (47.5%) patients. The median hematocrit level at admission was 40.7.  Table 2 summarizes the clinical presentation of PCR-positive dengue patients.

A total of 560/610 (91.8%) cases were diagnosed with DF, while 50 (8.2%) were diagnosed with DHF. The patients were reported with different signs and symptoms, including fever in 610 (100%) patients, followed by abdominal pain in 435 (71.3%), retro-orbital pain in 436 (71.5%), headache in 362 (59.3%), and hemorrhage in 252 (41.3%) patients. The clinical presentation was also compared according to disease severity. Abdominal pain and hemorrhages were significantly associated with DHF (*p* value <0.05). Similarly, severe thrombocytopenia was reported more commonly (*p* value <0.05) in DHF patients. Hematocrit, the hallmark of plasma leakage, was significantly higher in DHF patients (Table 2). No significant difference (*p* value >0.05) was observed among the age groups of the patients according to disease severity.

Type of occupation might play a significant role in the spread of vector-borne diseases in the communities. A total of 103 (16.9%) patients were construction laborers, 80 (13.1%) were students, 72 (11.8%) were healthcare workers, 61 (10%) were housewives, 56 (9.2%) were teachers, 49 (8.0%) were factory workers, 29 (4.8%) were engineers, 26 (4.3%) were IT workers, 25 (4.1%) were drivers, 25 (4.1%) were office workers, 23 (3.8%) were salesmen, and 16 (2.6%) were business people. Among them, 38 (6.2%) included retired persons, while 7 (1.1%) were unemployed.

## 4. Discussion

Currently, no specific antiviral agent is available for treating DENV infection; therefore, early diagnosis remains the mainstay for the timely management of dengue fever to prevent any complications and severity of the disease [16]. In the current study, we compared the diagnostic accuracy of ELISA to detect DENV NS1 antigen using qRT-PCR as a gold standard.

The current study demonstrated that DENV infections were more commonly detected in males (66.34%) than in females (33.65%). It may be due to the high ratio of outdoor working activities among males compared to females. A study in Uttar Pradesh, India, in 2020, by Kumar shows that the proportion of males is more than females (1.54 : 1), which supports our study [17]. Another study showed that males were more affected than females (1.5 : 1) due to their frequent outdoor activities [18].

The most affected age group in the recent study was 15 to 30 (41.1%) years old, followed by 31 to 45 (23.8%) years. Similarly, a study conducted in Pakistan in 2010 demonstrated that the age group of 20- to 30-year-olds was more affected by DENV infections than other age groups [19]. A retrospective study performed in China in 2020 showed a high prevalence among 31- to 45-year-old and 15- to 30-year-old age group, while a low incidence of dengue was seen in <14-year-old and >60-year-old age groups [20]. The highest prevalence of DENV infection in this particular age group was demonstrated by the fact that the highest proportion (63%) of the Pakistani population comprises youth aged 15 to 33 years [21].

Socioeconomic status plays a significant role in the transmission and distribution of DENV infections in the community [22]. Interestingly, the highest prevalence (16.9%) of DENV infections was reported among construction laborers, highlighting the public health significance of surveillance and insecticide spraying of buildings under construction and buildings not owned by the general public (like Government buildings). Construction sites are the ideal breeding sites for mosquitoes as water is stored in open plastic containers for longer durations, water pockets form during construction sites, there is higher relative humidity, and low light penetration is possible inside the buildings under construction. Moreover, excessive sweating during work also makes laborers more attractive to mosquitoes.

The prevalence of high DENV infections among students (13.1%) and teachers (9.2%) also identifies the failure of integrated vector management activities at public sector institutes.

Astonishingly, healthcare workers were the third-most affected group (11.8%) after the construction laborers and students. Healthcare professionals usually acquire DENV as a nosocomial infection during patient care and treatment, particularly in endemic areas [23]. One of the reasons could be their vicinity to the dengue patients, thus increasing the chances of human-to-human transmission during a mosquito bite. It also identifies the loopholes in the vector control measures at public sector hospitals and the carelessness on the part of the healthcare workers regarding the preventive measures to avoid mosquito bites.

Garages and motor vehicles are the other neglected sites where mosquitos might be found. Vehicles do not provide ideal breeding sites, but they serve as excellent blood-feeding sites as people (including children) often sit close to each other and are inactive. Among all others, drivers spend most of their time inside these vehicles and might get exposed to mosquito bites for the most extended duration. A sufficient number of dengue patients were drivers (4.1%) in this study.

The current study detected all four DENV serotypes circulating in Lahore, Pakistan, which is alarming as it might result in an outbreak of more severe DF. DENV-2 was the most prevalent (95.4%) serotype detected in dengue patients. Many extrinsic (viral) and intrinsic (host/physiological) factors play an essential role in the outcome of DENV infection [24]. DENV-2 was associated with severe outcomes of DF [24]. However, this study found no significant association between DENV serotypes and severe outcomes (DHF). Infections with multiple DENV serotypes are associated with the severe outcome of DF [25, 26]. However, no significant association with concurrent infection was noted in this study.

The current study also evaluated the diagnostic accuracy of ELISA NS1, taking qRT-PCR as a gold standard. ELISA NS1 is more widely available in Pakistan, while qRT-PCR is available only in specialized facilities. Therefore, it is significant to determine the diagnostic accuracy of the ELISA NS1 kit against a gold standard. NS1 antigen is a highly conserved glycoprotein in *Flaviviruses* that can be detected in the patient's serum as early as day one and usually remains detectable till day 9 of the onset of the symptoms. Thus, it serves as an important marker for the early diagnosis of DENV infection [27, 28]. ELISA is a powerful technique and is widely available worldwide due to its cost-effectiveness, reliability, and requirement of less sophisticated facilities compared to qRT-PCR.

In contrast, qRT-PCR is rapid, much more sensitive, and specific than ELISA [29]. The current study determined the low diagnostic accuracy of DENV NS1 ELISA (64.89%) by taking quantitative multiplex qRT-PCR as a gold standard. All the samples suspected of acute DF must also be tested by qRT-PCR for final confirmation. Another vital factor that could result in the degradation of the DENV RNA is unsuitable storage and transportation conditions, thus falsely resulting in a decline in the number of positive samples through qRT-PCR [30], thus affecting the diagnostic accuracy of the kit.

## 5. Conclusion

After analyzing the data, this study has identified a concerning situation of cocirculation of all four DENV serotypes in Lahore, with DENV-2 being the most common. Such a scenario could lead to a potential outbreak of a severe form of the disease. Thus, early detection of dengue fever is crucial to prevent complications, economic loss, and mortality. This study evaluated the diagnostic accuracy of the NS1 ELISA kit, revealing a sensitivity of 86.72% and a specificity of 43.61%. Additionally, our findings indicate that patients with severe thrombocytopenia and higher hematocrit levels are more susceptible to developing DHF. Notably, the study highlights the role of occupation in the spread of vector-borne diseases in communities, with construction laborers and healthcare workers being identified as high-risk groups for dengue virus infection in Lahore. Overall, these findings provide valuable insights for policymakers to develop targeted interventions to control the spread of dengue virus infection in Lahore, ultimately leading to improved public health outcomes.

## Figures and Tables

**Table 1:** Diagnostic accuracy of the DENV ELSA NS-1 antigen kit against qRT-PCR.

Statistic	Diagnostic accuracy (95% CI)
Sensitivity	86.72% (83.77% to 89.31%)
Specificity	43.61% (39.68% to 47.60%)
Positive predictive value	59.98% (58.15% to 61.78%)
Negative predictive value	77.12% (72.98% to 80.79%)
Accuracy	64.89% (62.15% to 67.55%)

**Table 2:** Demographics and clinical characteristics of dengue patients.

Characteristics	Total	DF	DHF	*p* value^*∗*^
	*N* (%)	*N* = 560 (%)	*N* = 50 (%)	
Median age (interquartile range)	30 (21–45)	30 (21–41)	35 (21–50)	0.487

*Gender*				
Male	415 (68)	387 (69.1)	28 (56)	0.08
Female	195 (32)	173 (30.9)	22 (44)	

*Clinical presentation*				
Fever	610 (100)	560 (100)	50 (100)	1.0
Myalgia/arthralgia	418 (68.5)	387 (69.1)	31 (62)	0.340
Abdominal pain	435 (71.3)	391 (69.8)	44 (88)	0.005
Retro-orbital pain	436 (71.5)	403 (72.0)	33 (66)	0.414
Headache	362 (59.3)	332 (59.3)	30 (60)	1.0
Hemorrhage	252 (41.3)	205 (36.6)	47 (94)	<0.0001

*Hematological parameters*				
Leukopenia	290 (47.5)	265 (47.3)	25 (50)	0.768
Thrombocytopenia (<150000) at presentation	548 (89.8)	498 (88.9)	50 (100)	0.006
<100,000	430 (70.5)	380 (67.9)	50 (100)	<0.001
<50,000	211 (34.6)	182 (32.5)	29 (58)	0.001
<25000	77 (12.6)	62 (11.1)	15 (30)	0.001
Hematocrit level at admission (median (SD))	40.7 (8.7)	41 (7.5)	50.9 (18.4)	0.014

## Data Availability

The data used to support the findings of this study are available from the corresponding author upon request.
